# An explanatory randomised controlled trial of a nurse-led, consultation-based intervention to support patients with adherence to taking glucose lowering medication for type 2 diabetes

**DOI:** 10.1186/1471-2296-13-30

**Published:** 2012-04-05

**Authors:** Andrew Farmer, Wendy Hardeman, Dyfrig Hughes, A Toby Prevost, Youngsuk Kim, Anthea Craven, Jason Oke, Sue Boase, Mary Selwood, Ian Kellar, Jonathan Graffy, Simon Griffin, Stephen Sutton, Ann-Louise Kinmonth

**Affiliations:** 1 Department of Primary Health Care Sciences, University of Oxford, Oxford, UK; 2 General Practice and Primary Care Research Unit, Department of Public Health and Primary Care, Institute of Public Health, University of Cambridge, Cambridge, UK; 3 Centre for Health Economics and Medicines Evaluation, Bangor University, Bangor, UK; 4 King’s College London, Department of Primary Care and Public Health Sciences, London, UK; 5 MRC Clinical Epidemiology Unit, Cambridge, UK

**Keywords:** Adherence, Brief intervention, Diabetes

## Abstract

**Background:**

Failure to take medication reduces the effectiveness of treatment leading to increased morbidity and mortality. We evaluated the efficacy of a consultation-based intervention to support objectively-assessed adherence to oral glucose lowering medication (OGLM) compared to usual care among people with type 2 diabetes.

**Methods:**

This was a parallel group randomised trial in adult patients with type 2 diabetes and HbA_1c_≥7.5% (58 mmol/mol), prescribed at least one OGLM. Participants were allocated to a clinic nurse delivered, innovative consultation-based intervention to strengthen patient motivation to take OGLM regularly and support medicine taking through action-plans, or to usual care. The primary outcome was the percentage of days on which the prescribed dose of medication was taken, measured objectively over 12 weeks with an electronic medication-monitoring device (TrackCap, Aardex, Switzerland). The primary analysis was intention-to-treat.

**Results:**

211 patients were randomised between July 1, 2006 and November 30, 2008 in 13 British general practices (primary care clinics). Primary outcome data were available for 194 participants (91.9%). Mean (sd) percentage of adherent days was 77.4% (26.3) in the intervention group and 69.0% (30.8) in standard care (mean difference between groups 8.4%, 95% confidence interval 0.2% to 16.7%, *p* = 0.044). There was no significant adverse impact on functional status or treatment satisfaction.

**Conclusions:**

This well-specified, theory based intervention delivered in a single session of 30 min in primary care increased objectively measured medication adherence, with no adverse effect on treatment satisfaction. These findings justify a definitive trial of this approach to improving medication adherence over a longer period of time, with clinical and cost-effectiveness outcomes to inform clinical practice.

**Trial registration:**

Current Controlled Trials ISRCTN30522359

## Background

Between a third and a half of medicines prescribed for long-term conditions are not taken as prescribed [1]. This applies equally to patients with type 2 diabetes who are managed with multiple medications to control cardiovascular risk factors and blood glucose [2]. Up to 37% of patients with diabetes have discontinued oral hypoglycaemic drugs within one year of initiating treatment [3], with adherence to medication falling as dosage frequency rises [4]. For those who persist with therapy, it is estimated that about 70-80% of doses are taken as prescribed [5].

Failure to take medication reduces the effectiveness of the treatment, and wastes healthcare resources in prescribed medicines not taken, extra consultations, referrals, investigations and hospital admissions [6,7]. The availability of an effective consultation-based intervention to support patients with long-term, progressive disorders in taking their medication regularly would make a major contribution to human health.

A variety of approaches to help patients take their medication regularly have been tested for efficacy [8]. However, there are only a few rigorous trials, and these suggest that interventions with multiple components are most effective in improving clinical outcomes [8]. A greater focus on the determinants of non-adherence may provide a basis for improved effectiveness, as interventions should address the principal causes of sub-optimal adherence. Since causes are many and vary between individuals, the intervention may need to be tailored to the individual.

The majority of studies have used measures of adherence that are imprecise, often relying on self-report [8]. This can lead to biased estimation of intervention effects, both within and between patient groups. Electronic measurement of adherence is increasingly used in intervention studies, but there few trials of its use among patients with type 2 diabetes have used it to date.

We have therefore drawn on psychological evidence and theory about hypothesised causes of non-adherence relating to weak motivation (intentional non-adherence) and forgetting (non-intentional non-adherence) [9], to develop a nurse-led consultation-based intervention (the “Support and Advice for Medication Study”; SAMS) targeting these hypothesised determinants through elicitation of personal beliefs. The intervention aims to increase patients’ motivation to take their tablets regularly by reinforcing positive beliefs and facilitating problem-solving around negative beliefs and to help patients translate motivation into action by asking them to form and write down specific action plans [10-12]. Both components are delivered in a single, brief intervention, although, if effective, future work could explore delivery over a longer period of time. We evaluated this new intervention in an explanatory randomised controlled trial to establish the short-term efficacy of the intervention on tablet taking behaviour and to inform estimates of the sample size for a pragmatic and definitive trial with glycaemic control as the outcome. The trial was carried out in a primary care setting among adults with type 2 diabetes taking oral glucose lowering medication (OGLM), with electronic measurement of medication taking.

## Methods

### Trial design

**Figure 1:**
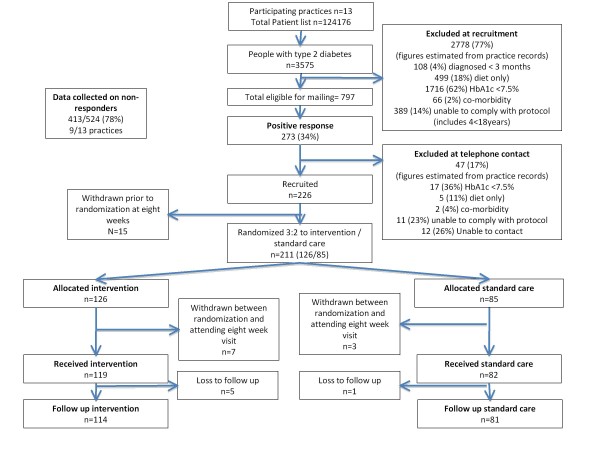
Trial Profile.

This trial formed part of a programme of work to evaluate the efficacy of a new intervention, and obtain information to refine its delivery and determine parameters for future trial evaluation. We used a parallel group trial design to evaluate a two-component intervention targeting motivation and using action planning in comparison with a control “standard care” intervention (Figure 1). An unbalanced (3:2) randomisation was used to provide more data from patients exposed to the new intervention, with little loss of power. The trial was carried out in weeks 9 to 20 of a 20-week study in which there was an initial, randomised evaluation of the impact of electronic medication measurement on adherence. In the initial phase of this study (weeks 1 to 8), patients were randomised to test the extent to which prior exposure to use of an electronic medication container might affect the results of the subsequent trial. Randomisation to the intervention and standard care groups of the efficacy trial took place at week 9 (see diagram).

The trial statistician randomly allocated patients independently of trial co-ordination and intervention delivery teams. A partial minimisation procedure was used to dynamically adjust randomisation probabilities to balance the baseline stratification variables. These included self-reported adherence, the baseline allocation to use or non-use of the electronic medicines measure and the baseline HbA_1c_. The London multi-centre research ethics committee reviewed and approved the protocol (06/MRE02/3).

### Setting and patients

Patients were recruited from 13 general practices (primary care clinics) in Oxfordshire, Buckinghamshire, Suffolk, Essex and Huntingdonshire (UK). Patients were eligible for inclusion if aged 18 years or over with type 2 diabetes of at least three months duration, able to give informed consent, currently taking any oral glucose-lowering agent and with a HbA_1c_ ≥7.5% (58mmol/mol). Patients were not excluded if taking insulin. Those approached were deemed by their general practitioner to be appropriate for tight glycaemic control and independent in medication taking.

### Trial measures

The primary outcome was the percentage of days over a twelve-week period on which the correct number of doses of main glucose lowering medication was taken each day as prescribed. It was measured using a validated measuring device [13], a container with a lid incorporating an electronic device that recorded the occurrence and timing of opening (TrackCap, Aardex, Zurich, Switzerland). A single treatment was tracked for each patient during the period of the trial, with metformin the preferred medication.

Secondary outcomes included: functional status measured with the 12-item Short Form Medical Outcomes Study health survey questionnaire (SF-12) [14], treatment satisfaction measured with the Diabetes Treatment Satisfaction Questionnaire (DTSQ) [15], satisfaction with communication with the nurse delivering the intervention and the Medication Adherence Report Scale (MARS) [16]. The MARS scale assesses adherence to medication with a five-item self report scale each with item responses scored on a five point scale. Scores are summed to give a score ranging from 5 to 25 with a higher score indicating higher levels of reported adherence. In addition, the number of medications taken was recorded and HbA_1c_ was measured in a central laboratory. The measures are fully described in the trial protocol [10].

### Trial intervention

Eight weeks after recruitment, patients were invited to the intervention visit to record and review their medication and randomised to either an intervention to support medication adherence or a standard care visit in which trial measurements were taken. The intervention had been developed and piloted after a detailed study to identify beliefs held by patients about diabetes and medication taking [17]. The intervention was delivered by a clinic nurse in each practice.

A clinical psychologist and intervention facilitator provided initial training for the clinic nurses at a day meeting supported by a detailed manual [10]. The nurses used protocols to standardise delivery of both the intervention and the standard care visit. The psychologist and intervention facilitators provided coaching and feedback to the nurses to ensure that the intervention and standard care were delivered as planned and to ensure intervention fidelity. This addressed possible sources of contamination in intervention delivery including the need to avoid (i) delivering the intervention to standard care patients, (ii) discussing motivational strategies and action planning with other members of the primary care team and (iii) using intervention strategies not specified in the protocol. Delivery of protocols was monitored by formal assessment of audio-taped consultations with all intervention participants and a sample of standard care participants [10].

In the first, motivational component of the intervention, the nurse elicited patients` beliefs relevant to their intention to take medication regularly as prescribed using a series of questions based on the Theory of Planned Behaviour [11]. These included perceived benefits and harms of taking medicines, views of other people who were important to them and factors that may facilitate or inhibit taking medicines regularly as prescribed. Positive beliefs were reinforced verbally and non-verbally through provision of tailored information and problem solving was facilitated around negative beliefs. In the second, action planning component, the nurse asked patients to generate and write down the exact circumstances in which they would take their medication (using an “if-then” formulation to elicit where, when and how this would occur) [12]. In the standard care visit, delivered by the same clinic nurses, none of the above techniques were applied.

### Study procedures

The clinic nurse identified eligible patients registered with the practice. Eligible patients were sent a letter from the practice giving details of the trial, and a questionnaire asking about basic demographics, medication regimen, medication adherence and beliefs about taking diabetes medicines. Responders were telephoned by the clinic nurse to arrange a recruitment visit to the full twenty week study period. Patients eligible and willing to take part were randomly allocated in advance of their recruitment visit to receive their medication in a medication monitoring device or in standard packaging.

At the 40-min trial recruitment visit with the clinic nurse, patients gave informed consent, including consent for tape-recording interviews for the purposes of training and assessment of fidelity of intervention delivery. Clinical data were collected, blood was taken, and questionnaires completed. For those patients allocated to the electronic medication-monitoring device, its use was explained, and the practice dispenser or pharmacist dispensed the patient’s usual prescription for metformin or alternative oral glucose lowering agent in the device. For those allocated to standard packaging, the practice dispenser or pharmacist provided medication in standard blister-packs. A follow-up and intervention visit was arranged after eight weeks.

In advance of the intervention visit, patients were sent a questionnaire from the coordinating centre. Also in advance of the intervention visit, patients were centrally randomised to the intervention or standard care to allow the clinic nurse to prepare to follow the allocated intervention schedule. At the visit, patients allocated to the intervention took part in a consultation, intended to last 50 min, with the clinic nurse that included the intervention (approximately 30 min) and data collection (approximately 20 min). The standard care visit lasted approximately 20 min during which study data were collected. At the intervention visit, blood samples were taken from all patients and enquiries were made about any possible adverse events including hypoglycaemia. All patients were dispensed their usual prescription for metformin or alternative oral glucose lowering agent in a medication monitoring device. A postal questionnaire was completed one week after the intervention visit. Final follow up for all patients at 20 weeks involved a visit to the clinic nurse and included retrieval of the medication monitoring devices, a blood sample for measurement of HbA_1c_ and a final questionnaire.

Resource use data were collected on the time taken by clinic nurses to deliver the intervention and collect clinical samples at the intervention visit (preparation time, duration of visit and other input) and, in the case of standard care, the time taken to collect clinical information. Data were also collected on the time taken by intervention facilitators to train the nurses and to provide feedback.

### Analysis methods

The trial was planned to follow up 200 patients, providing 80% power at the 5% significance level to detect a difference in means between randomised groups of 5% (1.5 days per month difference) in the percentage of days on which the correct number of doses was recorded as being taken. This was based on an estimate of the standard deviation of this measure of 13.5% in a pilot study for the trial conducted in 2001 in Newmarket, Cambridgeshire [10].

Analysis was by intention to treat and continuous outcomes were analysed adjusting for their corresponding baseline value, where this was measured, to improve precision. Where applicable, the missing indicator method was used [18], so that patients with a missing baseline value could be incorporated. Laboratory measurements and medication monitoring data were analysed blind to treatment allocation.

The primary outcome was adherence, defined as the percentage of days over 12 weeks on which the correct number of doses was taken. It was calculated from medication monitoring data recorded from the day after the intervention visit (week 9) following randomisation through to the day of the last visit (week 20). Mean adherence was compared between the intervention and control groups using the non-parametric percentile bootstrap method to derive the difference in means with a 95% confidence interval.

Subgroup analyses were carried out to explore the impact on the intervention effect of pre-specified baseline subgroup variables: HbA_1c_, age, gender, number of medications, self-reported adherence, and prior randomization to the electronic medication-monitoring device. These were assessed by testing the effect on the primary outcome of the interaction between each subgroup variable and the randomised group. For this purpose, continuous subgroup variables were dichotomised at the median. An additional analysis was carried out to explore the extent to which prior use of the medication-monitoring device affected nine to 20 week adherence.

## Results

In the 13 participating practices, 797 registered patients with type 2 diabetes potentially meeting the inclusion criteria were identified, of whom 273 responded as eligible, and 211 were confirmed as eligible on assessment and randomised with 92% follow up (Figure 1). Recruitment began in July 2006 and follow up was completed by November 2008. Trial participants in intervention and standard care groups were similar, in their early 60s with diabetes for less than 10 years and taking on average 6 medications daily and reporting a score of ≥24 on the MARS scale indicating high medication adherence. A slightly greater proportion of men were allocated to standard care (Table 1). In 9 of the 13 trial practices comparison of trial participants and non-responders suggested that trial participants had a shorter duration of diabetes and were more likely to be prescribed metformin (Table 2).

**Table 1:** Baseline characteristics of trial participants

	**Intervention arm**	**Standard Care arm**	**All participants**
			
**Socio demographic**	N=126	N=85	N=211
% male (N)	61.9% (78)	70.6% (60)	65.4% (138)
Age (years)	62.5 (11.0)	64.1 (10.3)	63.2 (10.7)
IMD Deprivation rank (0-100)	10.2 (6.4)	10.4 (6.8)	10.3 (6.8)
**Health-related**			
SF12 Physical [3] (norm 50	43.3 (11.2)	45.5 (10.3)	44.2 (10.9)
range 0-100)			
SF12 Mental [3] (norm 50	48.0 (10.6)	50.0 (9.6)	48.8 (10.2)
range 0-100)			
Duration of diabetes (years)	6.7 (4.8)	6.9 (5.3)	6.8 (5.0)
Weight (kg)	97.4 (21.7)	94.5 (19.6)	96.2 (20.9)
Systolic blood pressure (mmHg)	137.4 (16.2)	136.2 (15.9)	136.9 (16.0)
Diastolic blood pressure (mmHg)	78.3 (8.9)	78.1 (9.1)	78.2 (9.0)
HbA_1c_ (%) [1]	8.37 (1.25)	8.28 (1.22)	8.33 (1.24)
HbA1c (mmol/mol)	68.0 (2.7	67.0 (2.4)	67.5 (2.6)
**Medication-related**			
% treated with metformin (N) [4]	86.7% (104)	87.9% (73)	87.2% (177)
Metformin daily dose (mg) * [4]	1450 (795)	1525 (780)	1480 (788)
Total number of medications taken/day	5.7 (2.4)	5.9 (2.6)	5.8 (2.5)
Adherence (MARS) [2] (range 5-25)	23.6 (2.3)	23.6 (2.8)	23.6 (2.5)

Values are mean (SD) unless otherwise stated: missing data: [1] 17; [2] 23; [3] 2; [4] 8.

* for those treated with metformin.

MARS Medication Adherence Report Scale.

**Table 2:** Comparison of non-responders with trial participants*

	Participants*	**Non-Responders**
	**Mean (SD)**	**Mean (SD)**
	N = 133*	*N = 413*
% male (N)	65.4 (87)	62.7 (259)
Age (years)	62.7 (10.7)	64.7 (12.8)
HbA_1c_ (%)	8.3 (1.2) [1]	8.7 (1.5) [2]
HbA1c (mmol/mol)	67 (13)	72 (16)
Duration of diabetes (years)	6.5 (4.9)	8.1 (7.1) [3] **
% prescribed metformin (N)	87.8 (115) [4]	76.0 (307) [5] **

Values are mean (SD) unless otherwise stated.

Missing data: [1]n = 9; [2]n = 1; [3]n = 5; [4]n = 2; [5]n = 5.

*Those participants from the nine practices in which non-responder data were collected.

** *P* < 0.05.

Patients allocated to the intervention took their prescribed number of doses of medication on a significantly higher percentage of days compared to the standard care group (Table 3). The mean difference between groups in percentage of days that the correct number of doses of medication was taken as prescribed was 8.4% (95% confidence interval 0.2% to 16.7%, p = 0.044). No patients were identified as stopping their medication during the period of twelve weeks. There were no significant differences between groups for secondary outcomes including self report medication adherence (MARS), SF-12, diabetes treatment satisfaction, HbA_1c_, satisfaction with communication with the nurse, or hypoglycaemia (Table 3).

**Table 3:** Outcomes twelve weeks after randomisation to intervention or standard care

	**Intervention arm**		**Standard care arm**		**Intervention effect (95% C.I)**	**P-value**
	**Baseline**	**Final visit**	**Baseline**	**Final visit**		
**Primary outcome**						
Days correct dose taken (SD) (%)*	-	77.4 (26.3)^a^		69.0 (30.8)^b^	8.4 (0.2, 16.7) ^c^	0.044
**Secondary outcomes**						
SF12 Physical (SD) ^†^	43.3 (11.2) ^r^	44.6 (11.1) ^c^	45.5 (10.3) ^r^	46.3 (9.0) ^l^	-0.7 (-2.7, 1.4) ^s^	0.52
SF12 Mental (SD) ^†^	48.0 (10.6)	49.5 (10.4) ^c^	50.0 (9.63) ^t^	52.6 (8.8)^l^	-1.6 (-3.9, 0.6) ^s^	0.15
Diabetes treatment satisfaction (SD)	-	30.6 (5.4) ^c^	-	31.3 (4.6) ^f^	-0.7 (-2.2, 0.7) ^u^	0.32
HbA_1c_ % (SD) ^†^	8.34 (1.26) ^k^	8.34 (1.24) ^k^	8.29 (1.23) ^l^	8.21 (1.32) ^f^	0.06 (-0.19, 0.32) ^m^	0.64
HbA_1c_ mmol/l (SD)	67.7 (13.8)	67.7 (13.6)	67.1(13.4)	66.2 (14.4)	0.7 (- 2.1, 3.5)	
**Medication-related**						
MARS Self report adherence (SD) ^†^	23.6 (2.3) ^d^	23.6 (2.6) ^e^	23.6 (2.8) ^f^	24.1 (1.6) ^g^	-0.4 (-1.0, 0.2) ^h^	0.20
Satisfaction with communication (median, IQR)**		4 (2,5)		4 (2,5)		0.13
% reporting hypoglycaemia (N)***		1.6% (2)		0,0 (0)		0.52*

Values are mean (SD) unless otherwise stated. Missing values: a = 13, b = 4, c = 17, d = 15, e = 27, f = 8, g = 12, h = 39, k = 16, l = 9, m = 24, r = 1, s = 26, t = 2, u = 25.

* - Estimate of intervention effect adjusted for baseline where available and 95% confidence interval derived from the non-parametric bootstrap percentile method with 50,000 replications. † = In the analysis of the final visit measure adjusting for a baseline that has occasional missing data, the missing indicator method is used. ** p value from Mann–Whitney test., *** p value from Fisher’s exact test. MARS Medication Adherence Report Scale.

There were no significant interactions to indicate that the effect of the intervention on the medication adherence outcome varied greatly by gender, number of medications, self-reported adherence (Table 4). However, taking into account the reported confidence intervals, the intervention effect may have been larger in those with better glycaemic control (*p* = 0.07), older age (*p* = 0.19) and higher self-reported adherence at baseline (*p* = 0.11). There was no effect of prior allocation of patients to the electronic medication-monitoring device in the first phase of the study with a mean (95% confidence intervals) difference of 6.0 (-10.5, 22.7) in percentage of days of medication not taken between groups (*p* = 0.48) (Table 4). In addition there was not an effect of prior allocation to the electronic medication monitoring device on overall nine to 20 week adherence (*p* = 0.84).

**Table 4:** Subgroup analysis of the intervention effect on the percentage of days adherence to prescribed medication

**Sub-group variable**	**Sub-group category (L = Low or H = High)**	**N per sub group**	**Intervention arm**	**Standard care arm**	Intervention- Control (SE)*	**Difference (H-L) (95% C.I.)**	**P interaction**
HbA_1c_ % (mmol/mol)	7.5 – 7.9 (58-63)	85	80.4 (24.5)	62.6 (34.2)	17.7 (6.4)		
	8.0 - 12.4 (64-112)	108	74.8 (27.7)	72.8 (27.8)	2.0 (5.3)	-15.8 (-32.9, 1.2)	0.07
Age (years)	37 – 64	94	75.0 (25.61)	72.3 (27.3)	2.7 (5.6)		
	65 and over	100	80.0 (27.1)	66.4 (33.2)	13.5 (6.0)	10.8 (-5.4, 27.2)	0.19
Total number of medications	0 – 5	89	80.2 (23.3)	73.0 (27.0)	7.2 (5.3)		
	6 and over	105	75.1 (28.5)	65 .2 (33.7)	10.0 (6.1)	2.8 (-13.5, 19.1)	0.74
Self reported adherence at baseline	0-23	102	75.0 (26.4)	70.9 (27.2)	4.1 (5.5)		
	24 or 25	70	85.9 (21.3)	68.0 (33.9)	17.9 (6.8)	13.8 (-3.2, 30.0)	0.11
Prior randomisation to medication monitoring device	No	102	76.6 (28.7)	71.0 (29.3)	5.6 (5.8)		
	Yes	92	78.2 (23.7)	66.65 (32.6)	11.6 (5.9)	6.0 (-10.5, 22.7)	0.48
Gender	Male	127	77.0 (28.3)	66.4 (31.5)	10.6 (5.3)		
	Female	67	78.0 (22.9)	74.8 (28.8)	3.3 (6.4)	-7.3 (-24.3, 9.6)	0.39

Effect estimates, confidence intervals and p-values from non-parametric bootstrap percentile method (50,000 replications).

* Estimated intervention effects are positive within each subgroup.

The mean total time (95% confidence interval) spent in delivering the intervention and associated clinical care data collection was 74 min (68 to 79) for the intervention group and 42 min (39 to 47) for the standard care group; a mean difference of 31 min (95% confidence interval 25 to 37). Intervention facilitators spent, on average, 2.3 h per patient listening to tape recordings, training nurses, and providing feedback in the intervention group, compared with 1.2 h in standard care.

## Discussion

A theoretically based, single session intervention delivered to patients with type 2 diabetes in primary care consultations by clinic nurses was effective in improving objectively measured glucose lowering medication adherence compared with standard care. The effect was seen consistently over the 12 weeks of the study. The intervention had no adverse effect on measures of functional status, satisfaction, communication or hypoglycaemia. There was no effect on glycaemia measured by HbA_1c,_ but the power and time frame of our trial were not designed to test for this effect.

This study addressed key weaknesses in previous studies that have limited the quality of the evidence concerning medication adherence. The most effective interventions to improve medication adherence and clinical outcomes have been complex, multi-component and intensive, but few studies have been designed to allow exploration of the reasons for success or failure of interventions and their delivery [19]. Target groups have often been poorly defined and characterised and trial participants also tend to be unusually adherent, limiting discovery of effects that would be important in general populations [20-22]. The most commonly used measures are self report and these are often associated with larger effects than objective measurement [23,24]. Trial designs themselves have often been weak with lack of attention to central randomisation and sources of bias. Not surprisingly, previous studies in this field have shown inconsistent effects.

We addressed these issues in the following ways. We developed the intervention systematically from psychological evidence and theory [25]. It is predicated on addressing weak motivation associated with ambivalence to medication taking [26], and the gap between intention and action that may be bridged by making specific action plans [11,12]. We ensured the intervention was feasible to deliver in a health service context and addressed quality assurance. Nurses were trained to deliver the intervention in workshops using scripts and feedback, and consultations were audio-taped to assure delivery as planned and to support a consistent approach to delivery across nurses over time.

We also identified a patient group with the potential to benefit from improving their adherence to medication. They comprised a well characterised population which reflected the kind of patients with diabetes seen in primary care every day: in their sixties, with established diabetes of seven years mean duration, and prescribed an average six of medications daily, including metformin, without having reached optimum glycaemic control. In addition we obtained an acceptable rate of participation from eligible patients, and were able to demonstrate that the characteristics of these individuals were similar to the wider population from which they were recruited.

We measured the primary outcome objectively using a validated electronic medication monitor [13] that allows a day-by-day description of adherence as well as providing summary measures. In an initial, randomised evaluation of the impact of electronic medication measurement on adherence we found that prior use of an electronic medication-monitoring device had no statistically significant effect either on adherence or in modifying the intervention effect on adherence for the primary outcome. The study design was rigorous with central randomisation and blinding of group allocation from those assessing outcome. Randomly allocated groups were well matched on the measured variables.

There are number of limitations to this study. The participation rate was not high, although attempts were made to mitigate this by anonymised collection of data on non-participants. We restricted our intervention to glucose lowering medication and excluded those in whom tight glucose control was inappropriate, although support for taking other medications might be appropriate for this group of patients. Detailed work was undertaken to minimise the possibility of contamination between intervention and usual care groups, although any failure of the procedures put in place would reduce the size of effect observed. Self reported adherence, as measured by the MARS self-report questionnaire, did not differ between intervention and usual care groups. MARS attempts to capture awareness of non-adherence due to forgetting, altering, stopping, missing or taking less medication than prescribed. Moreover, estimates of adherence were close to maximal in both groups as has been found elsewhere [17,27]. Thus, while the MARS results did not confirm our principal outcome of electronic monitoring, it may be because the latter is measuring a different component of adherence, being more sensitive to unconscious non-adherence, and also that it is less constrained by ceiling effects. Self-report measures, when used in trials may also be difficult to interpret, as they are more susceptible to outcome preference bias.

Our aim in this short-term explanatory study was to estimate the efficacy of the intervention on the behaviour of taking medication and the trial was therefore not powered to evaluate glycaemic effects. However, we anticipated that with a large effect on tablet taking, we might see some indication of an effect on HbA_1c_. Efficacy studies with similar time periods and doses of metformin (1500g per day) have demonstrated improvements in HbA_1c_ of around 1.5% compared with placebo among patients with very poorly controlled diabetes at baseline [28], but smaller effects among patients under better control [29]. However, the impact of the improved adherence of about one week over the three month period in our intervention group was not sufficient to alter overall glycaemia.

## Conclusions

We have demonstrated that a well-specified and reproducible consultation-based intervention delivered in a single session by clinic nurses in primary care can increase objectively measured medication adherence with no adverse effect on treatment satisfaction. Application of this approach offers the potential for reducing the burden of disease in diabetes managed by long-term medication. A larger pragmatic trial, with further development of intervention components, designed to sustain effect, with longer follow up, and powered to evaluate the effect of the intervention on clinical outcomes is justified.

## Competing interests

No potential conflicts of interest have been reported for this paper.

## Authors’ contributions

A.F. wrote the article, contributed to discussion and researched data. A-L.K. wrote the article, contributed to discussion and researched data. W.H. contributed to discussion, researched data and reviewed and edited the article. S.G. contributed to discussion, researched data and reviewed and edited the article. A.T.P. contributed to discussion, researched data and reviewed and edited the article. S.S. contributed to discussion, researched data and reviewed and edited the article. D.H. contributed to discussion, researched data and reviewed and edited the article. A.C. researched data and reviewed and edited the article. S.B. contributed to discussion, researched data and reviewed and edited the article. J.G. contributed to discussion, researched data and reviewed and edited the article. I.K. contributed to discussion, researched data and reviewed and edited the article. J.O. researched data. M.S. researched data and reviewed and edited the article. Y.K. researched data and reviewed and edited the article. All authors approved the final draft of the manuscript.

## Pre-publication history

The pre-publication history for this paper can be accessed here:

http://www.biomedcentral.com/1471-2296/13/30/prepub
